# AMPK‐upregulated microRNA‐708 plays as a suppressor of cellular senescence and aging via downregulating disabled‐2 and mTORC1 activation

**DOI:** 10.1002/mco2.475

**Published:** 2024-03-09

**Authors:** Jian Zhang, Hui Gong, Tingting Zhao, Weitong Xu, Honghan Chen, Tiepeng Li, Yu Yang, Ming Yang, Ning Huang, Chuhui Gong, Fangfang Wang, Cuiying Zhang, Jin Liu, Hengyi Xiao

**Affiliations:** ^1^ The Department of Aging and Geriatric Medicine National Clinical Research Center for Geriatrics State Key Laboratory of Biotherapy West China Hospital Sichuan University Chengdu China; ^2^ Department of Biochemistry and Molecular Biology and Molecular Medicine and Cancer Research Center College of Basic Medical Sciences Chongqing Medical University Chongqing China; ^3^ Department of Laboratory Medicine The Second People's Hospital of Changzhi City Changzhi China

**Keywords:** aging, AMP‐activated protein kinase (AMPK), Disabled 2 (DAB2), miR‐708, mammalian traget of rapamycin coplex 1 (mTORC1)

## Abstract

Senescence‐associated microRNAs (SA‐miRNAs) are important molecules for aging regulation. While many aging‐promoting SA‐miRNAs have been identified, confirmed aging‐suppressive SA‐miRNAs are rare, that impeded our full understanding on aging regulation. In this study, we verified that miR‐708 expression is decreased in senescent cells and aged tissues and revealed that miR‐708 overexpression can alleviate cellular senescence and aging performance. About the molecular cascade carrying the aging suppressive action of miR‐708, we unraveled that miR‐708 directly targets the 3′UTR of the disabled 2 (*Dab2*) gene and inhibits the expression of DAB2. Interestingly, miR‐708‐caused DAB2 downregulation blocks the aberrant mammalian target of rapamycin complex 1 (mTORC1) activation, a driving metabolic event for senescence progression, and restores the impaired autophagy, a downstream event of aberrant mTORC1 activation. We also found that AMP‐activated protein kinase (AMPK) activation can upregulate miR‐708 via the elevation of DICER expression, and miR‐708 inhibitor is able to blunt the antiaging effect of AMPK. In summary, this study characterized miR‐708 as an aging‐suppressive SA‐miRNA for the first time and uncovered a new signaling cascade, in which miR‐708 links the DAB2/mTOR axis and AMPK/DICER axis together. These findings not only demonstrate the potential role of miR‐708 in aging regulation, but also expand the signaling network connecting AMPK and mTORC1.

## Graphical abstract

Schematic depicting the AMPK prevents senescence via miR‐708‐mediated signaling cascade. Oxidative stress decreases the expression of miR‐708, but AMPK activation promotes miR‐708 maturation in a DICER‐dependent manner. miR‐708 directly targets the *Dab2* 3′‐UTR to reduce DAB2 expression and mTORC1 activity and autophagy inhibition, ultimately suppresses cellular senescence and mouse aging.

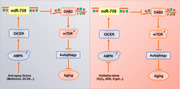

## 1 INTRODUCTION

As a universal feature of higher organisms, aging can develop at all levels in bodies, through cells to tissues and organs.^1^ Cellular senescence is the foundation of individual aging, featuring as cell cycle arrest, senescence‐associated secretory phenotype (SASP), metabolic disorders, autophagy/mitophagy dysfunction, and etc.^2^ Importantly, the accumulation of senescent cells in organisms is closely related to the occurrence of aging and age‐related diseases.^3^ Therefore, intensively exploring the molecular mechanism of aging at the cellular level is essential for understanding the occurrence and development of aging and exploiting valid antiaging strategies.

Many studies have investigated the expression patterns and the regulatory pathways of age‐related proteins. The core events which facilitate cellular senescence include cell cycle arrest, increment in proinflammatory cytokines expression (SASP), and metabolic disorder.^4^, ^5^, ^6^ Among the regulatory pathways connected with senescent/aging‐progression, the mammalian target of rapamycin complex 1(mTORC1) pathway and the AMP‐activated protein kinase (AMPK） pathway are two major ones, as both act crucially for cellular homeostasis. The mTOR pathway plays a central role in cell proliferation and survival by governing anabolism processes, meanwhile its activation also inhibits autophagy‐mediated catabolism.^7^ The AMPK pathway works for energy homeostasis, and its activation promotes autophagy‐mediated catabolism by multiple mechanisms including inhibiting the mTOR pathway.^8^ Despite that AMPK can suppress mTORC1 by phosphorylating TSC1, the relationship between AMPK‐driven miRNA expression and mTORC1 activity remains largely unknown. Of particularly importance from our view, increasing evidence have revealed that sustained mTOR activation promotes aging ^9^ and AMPK activation alleviates aging in various contexts,^10^ so that elucidating the microRNA (miRNA)‐bridged connections between mTOR and AMPK in aging context is worthy to be challenged.

In recent decades, miRNA‐involved posttranscriptional regulation on the expression of aging associated genes attracted increasing attentions. miRNA‐mediated gene expression also opened a new door to excavate the molecular matters involving aging regulation, that basically associate with the RNA stability and the protein translation of miRNA‐targeted genes, with different focus from traditional studies which largely aimed at matters of the sequence variation and transcriptional regulation of aging related genes, or the degradation and modification of aging‐related proteins. At present, miRNA molecules closely implicated with aging are collectively referred to as Senescence‐associated microRNAs (SA‐miRNAs).^11^, ^12^ The basic feature of SA‐miRNAs is the correlation of their expression with aging. SA‐miRNAs can be divided into two categories according to their function, we named them as aging‐promoting SA‐miRNAs and aging‐suppressive SA‐miRNAs. Nowadays, several dozens of aging‐promoting SA‐miRNAs have been identified, such as miR‐21, miR‐22, and miR‐34a,^13^, ^14^, ^15^ but only a few of aging‐suppressive SA‐miRNA are known, they are miR‐15b^16^ and miR‐106b.^17^ It means we have limited information about if and how miRNAs negatively regulate aging.

This study aims to discover a new aging‐suppressive SA‐miRNA and analyze its working mechanism, with special attention on the connection between mTOR and AMPK pathways. It includes the establishment of a SA‐miRNA profile of senescent cells, the clarification of miR‐708 as an aging‐suppressive SA‐miRNA, the identification of *disabled(Dab2)* as a direct target of miR‐708, the confirmation about the positive relationship of DAB2 with the mTORC1 pathway, and the regulatory effect of AMPK on miR‐708 expression. Both cellular and mouse experiments are included. Although this work exposed a new molecular signaling connecting mTORC1 and AMPK and identified a novel member of aging‐suppressive SA‐miRNA, miR‐708, further investigation needs to be engaged for creating antiaging approaches which work by targeting molecules functioning in this signaling.

## 2 RESULTS

### 2.1 miR‐708 expression is lower in senescent cells and aged tissues

**FIGURE 1:**
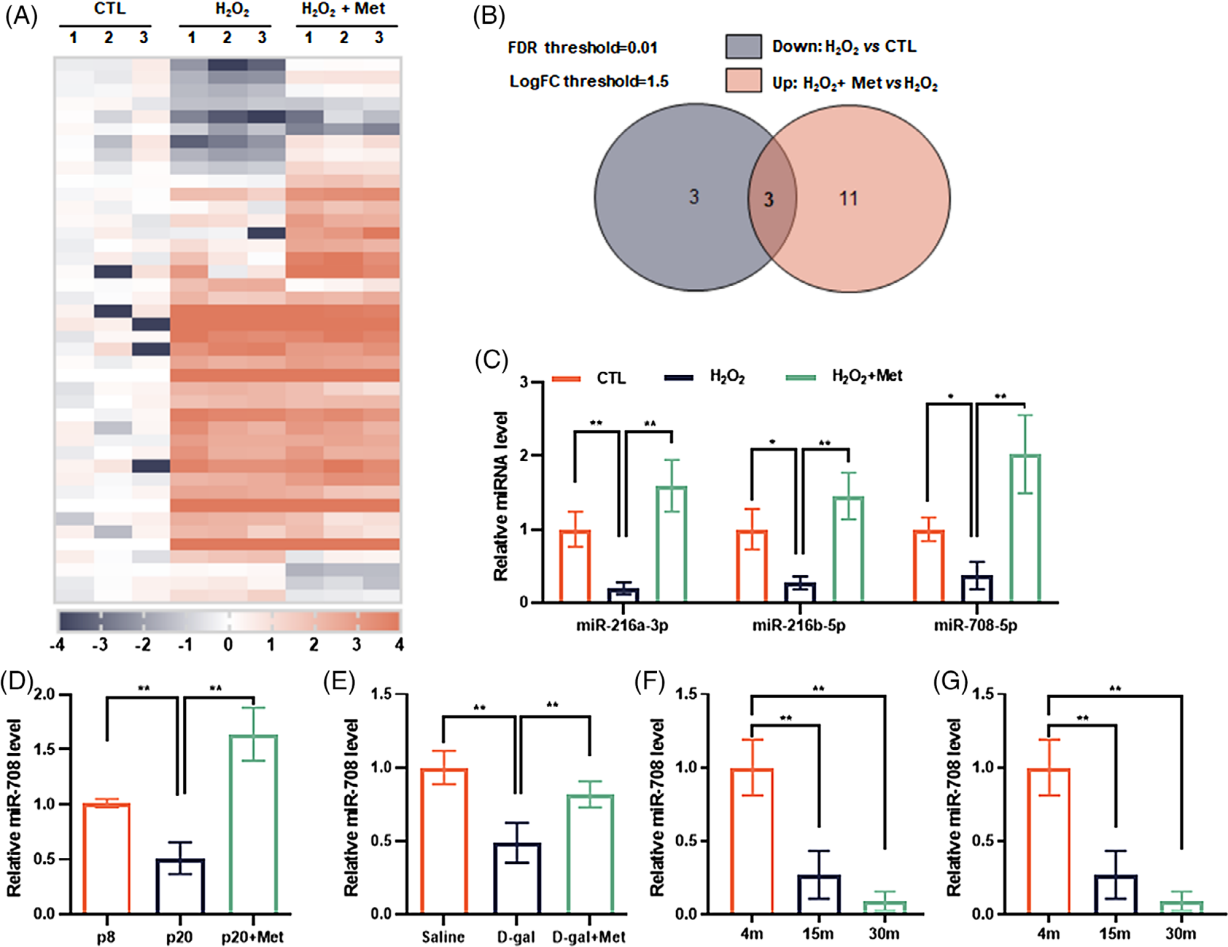
miR‐708 expression is negatively correlated with aging. (A) Heat map of small‐RNA‐sequencing of NIH3T3 cells. CTL: proliferating cells; H_2_O_2_: senescent cells, H_2_O_2_+Met: cells received H_2_O_2_ plus Met treatment (*n* = 3). (B) Venn diagram showing differentially expressed miRNAs (threshold fold change ≥1.5 and *p *< 0.01). (C) Relative level of miRNAs selected from the intersection of Venn diagram; NIH3T3 cells were employed and assessed (*n* = 3). (D) Relative miR‐708 level in human primary fibroblasts; cells of passage 8th, passage 20^th^, and Met‐treated passage 20th were employed and analyzed (*n* = 3). (E) Relative miR‐708 level in liver of mice with or without D‐gal‐ or Met‐injection (*n* = 3). (F) Relative miR‐708 level in brain of mice, aged at 4, 15, or 30 months (*n* = 3). (G) miR‐708 expression human blood with indicated ranges of age (*n* = 6). *p < 0.05, **p < 0.01.

In order to screen out new SA‐miRNAs that functionally associate with aging, sub‐lethal dose of hydrogen peroxide (H_2_O_2_)‐induced senescence cell model was used, because previous studies have evidenced the rationality of this model for stress‐induced premature senescence (SIPS) model, with the most senescent characters like those seen in replicative senescent cells.^18^, ^19^, ^20^, ^21^ Not only, metformin (Met), an AMPK pathway activator, was selected for senescence intervention.^18^, ^19^, ^22^ As shown in Figures S1A–C, H_2_O_2_‐induced senescent cells own the high level of p16 and p21 proteins, high rate of senescence‐associated β‐galactosidase (SA‐β‐gal) positive cells, and the elevated SASP production (mRNA level of *Il‐6* and *Il‐8*), while these senescence markers become blunt in Met‐treated cells. Based on this characterization, H_2_O_2_‐induced senescent cells, Met‐treated cells, and control proliferating cells were collected in parallel and sampled for small RNA sequencing (Figure 1A). Through analyzing the differential expression profile of miRNAs, it was found that six kinds of murine miRNAs were apparently less expressed in senescent cells, and fourteen kinds of miRNAs evidently more expressed in Met‐treated cells (Figure 1B). And, among these two sets of miRNAs, three were overlapping (Figure 1B) and this finding got confirmed by quantitative real‐time PCR (qPCR) experiments (Figure 1C). Based on literature retrieval^23^, ^24^ and preliminary experiments (Figures S2A–C), we focused further investigation on elucidating the aging implication of mmu‐miR‐708‐5p (hereinafter referred to as miR‐708).

The expression pattern of miR‐708 was examined also in replicative human senescent cells. Like results obtained using H_2_O_2_‐induced murine senescent cells, miR‐708 level in passage 20th cells was lower than that in passage 8th cells, whereas miR‐708 level in passage 20th cells with Met treatment was higher than that in passage 20th cells without Met treatment (Figure 1D). Accordingly, the signs of senescence altered inversely with miR‐708 level (Figures 1D and S1D, E).

Three evidence from in vivo experiments were also obtained. The first one came from the experiments using D‐galactose (D‐gal) stress‐induced aging‐like mice model.^25^, ^26^, ^27^ As shown, the miR‐708 expression in tissues (liver, brain and kidney) of aging‐like mice was less than that of control mice, but this expression increased by Met‐intervention (Figures 1E and S3A, B). The second evidence obtained from naturally aged mice, where the miR‐708 expression in the brain of aged mice was lower than that of young mice (Figure 1F). The third evidence got by detecting human blood sample. It shows that miR‐708 abundance in human blood declined significantly with age (Figure 1G). These findings together suggest that miR‐708 expression in cells and tissues, either mice or human, is downregulated with aging.

### 2.2 miR‐708 alleviates cellular senescence

**FIGURE 2:**
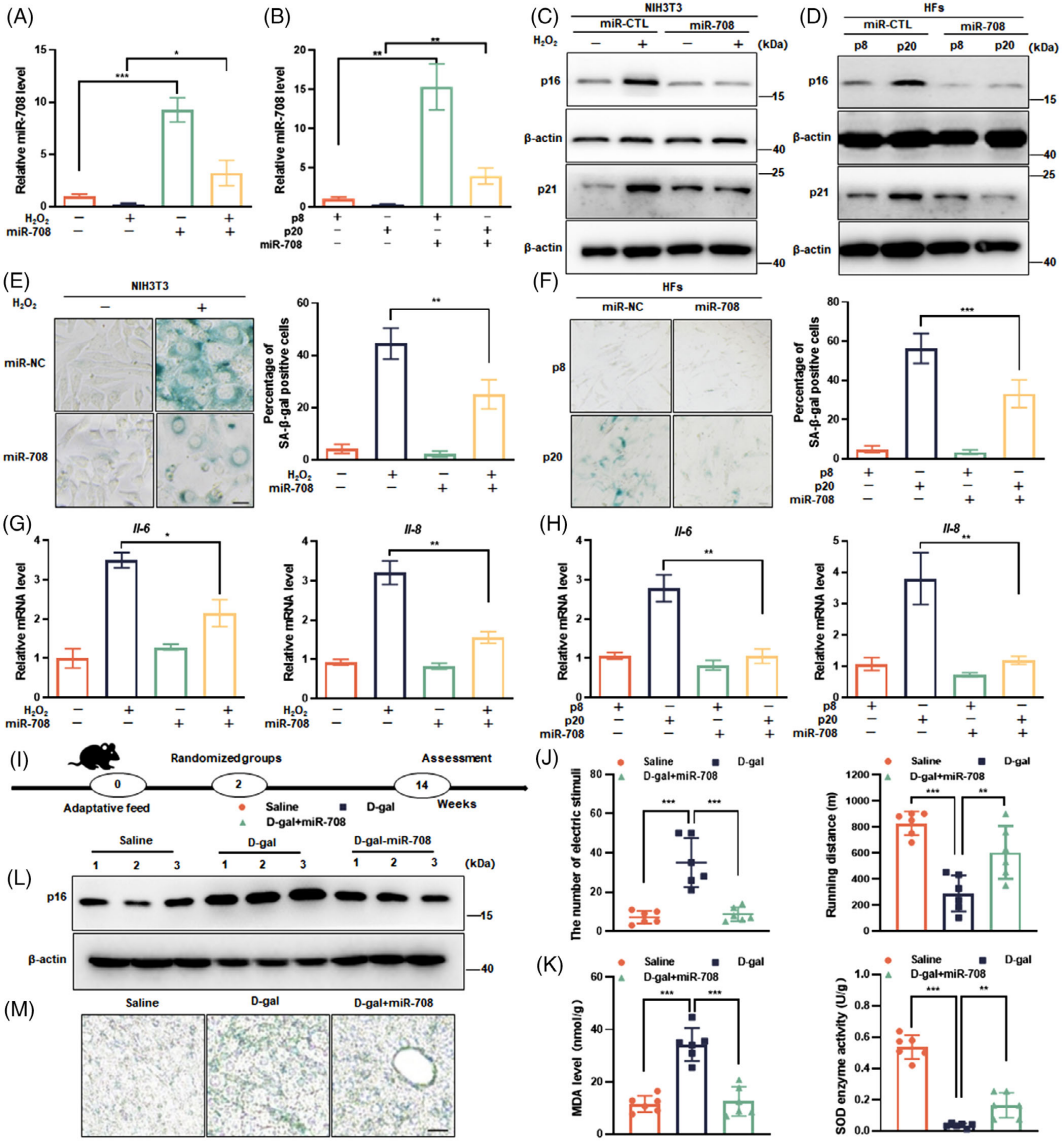
miR‐708 overexpression prevents aging. A, C, E, G: NIH3T3 cells were transfected with miR‐708 mimics or scrambled miRNA (miR‐CTL) for 24 h, followed by the senescence induction with H_2_O_2_. B, D, F, and H: Human primary fibroblasts cells (HFs) of passage 8 were transduced with lentivirus‐expressing miR‐708 or miR‐CTL, followed by cultivating and continuously passaging until passage 20. Cells at passage 8 and passage 20 were harvested for assays. (A and B) Relative miR‐708 level. (C and D) Representative images are shown by immunoblotting assays against p16 and p21 proteins. (E and F) Representative images are shown SA‐β‐gal staining (left), and average percentages of SA‐β‐gal positive cells (right). (G and H) Relative mRNA level of *Il‐6* and *Il‐8*. (I) The experimental procedure (*n* = 6). (J) Electric stimuli times (left) and running distance (right), recorded by treadmill. (K) MDA level (left) and SOD activity (right) in liver. (L) Representative images are shown by immunoblotting assays against p16 protein in liver. (M) Representative images are shown SA‐β‐gal staining of liver sections. Scale bar indicates 50 µm. All statistical data for cell culture experiments were calculated based on three independent experiments. *p < 0.05, **p < 0.01, ***p < 0.001.

To evaluate the causal correlation of miR‐708 with senescence, an overexpression strategy was adopted by transfecting synthetic miR‐708 mimics into cells (Figure 2A). As shown, compared with scrambled miRNA, miR‐708 mimics transfection caused the decrease in p16 and p21 protein level in H_2_O_2_‐treated cells (Figure 2C), the extent of SA‐β‐gal staining (Figure 2E), and the mRNA level of *Il‐6* and *Il‐8* (Figure 2G). To confirm this result, adriamycin (ADR)‐induced senescence model, another commonly used SIPS model^28^ was utilized. As expected, similar results were obtained (Figures S4A and B). Not only, consistent observation collected by using human embryonic lung fibroblast cell line (MRC‐5) (Figures S4C and D). Furthermore, we tested the influence of miR‐708 on the progression of replicative cellular senescence, by using human primary fibroblasts and lentivirus transduction for miR‐708 overexpression (Figure 2B). As shown, the increment of both p16 and p21 protein level, SA‐β‐gal staining and the mRNA level of *Il‐6* and *Il‐8* in senescent cells got alleviated under miR‐708 overexpression (Figures 2D, F, and H). Collectively, these results suggest that miR‐708 overexpression is protective against cellular senescence.

### 2.3 miR‐708 restrains mouse aging

Next, adeno‐associated virus serotype 9(AAV9)‐miR‐708 used in vivo experiments were engaged and the experimental procedure shown in Figure 2I. At first, AAV9‐mediated miR‐708 overexpression was ensured, showing as AAV9‐miR‐708‐green fluorescent protein (GFP) transduction brought a fluorescent signal in different tissues and organs with the strongest fluorescence the increased miR‐708 expression in liver (Figures S4E and F). Then, aging‐related performances were examined. The first set of examinations is physical strength, evaluating by behavior tests. As shown, D‐gal mice got more electric shocks and accomplished shorter running distance on treadmill machine, compared with saline‐treated control mice. Not surprisingly, the performance of the D‐gal+miR‐708 mice behaved remarkably better than that of D‐gal mice (Figure 2J). Consistent results were obtained from grip and memory tests, showing as the decreased forelimb grip power and spontaneous alteration in D‐gal mice comparing with saline‐treated mice, and the increased these parameters in D‐gal+miR‐708 mice compared with D‐gal mice (Figures S4G and H). This second set of examinations targets organ function and stress response. Results showed that D‐gal treatment caused the elevation of alanine aminotransaminase (ALTL) and aspartate aminotransferase (ASTL) activities (Figure S2I), a high level of malondialdehyde(MDA), whereas miR‐708 overexpression relieved these abnormalities. Accordingly, superoxide dismutase (SOD) activity in liver was altered inversely (Figure 2K). The third set of examinations focuses on the two most accepted molecular markers for aging, the p16 expression and the SA‐β‐gal staining. As shown, miR‐708 overexpression significantly decreased the abundance of p16 protein (Figure 2L) and the staining of SA‐β‐gal in mouse liver (Figure 2M), compared with D‐gal treatment alone.

Collectively, AAV9‐medicated miR‐708 overexpression alleviates aging‐like performances of D‐gal mice, with the eased D‐gal stress and oxidative damage in tissues and organs

### 2.4 *Dab2* is a direct target of miR‐708

**FIGURE 3:**
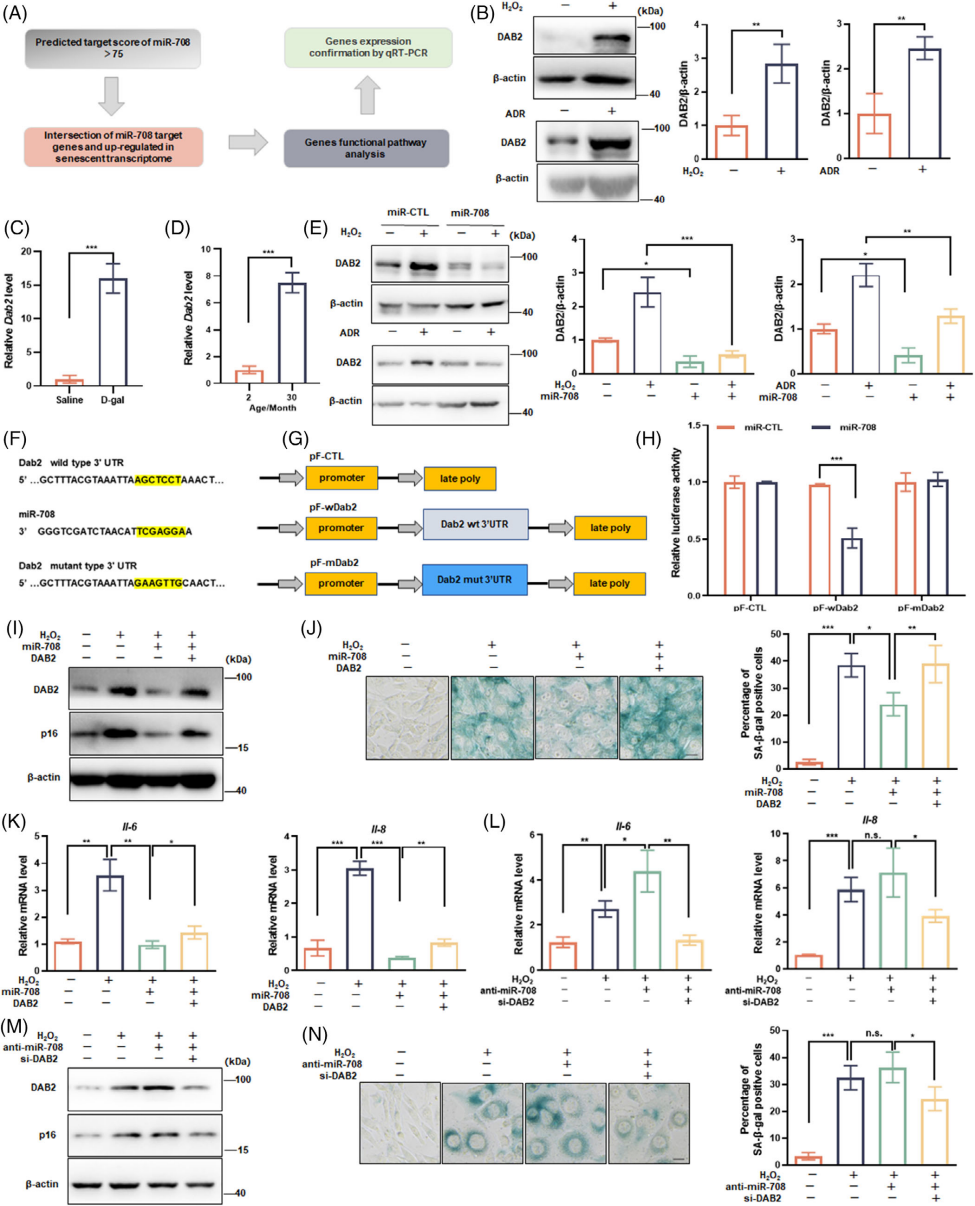
*Dab2* is directly targeted by miR‐708. (A) The procedure for screening target genes of miR‐708. (B) DAB2 protein levels in proliferating and H_2_O_2_‐ or ADR‐induced senescent cells were assessed by immunoblotting assays. Representative images from immunoblot assays (left) and the relative level of DAB2 protein normalized by β‐actin level were shown (right). (C and D) Relative mRNA levels of *Dab2* in the liver of D‐gal‐induced aging‐like mice or in the brain of natural aging mice are shown. (E) Cells transfected with miR‐708 mimics or scramble miRNA (miR‐CTL) received H_2_O_2_‐ or ADR‐senescence induction treatment. Representative images immunoblot assay are shown (left) and the relative level of DAB2 protein normalized by β‐actin level were shown (right). (F) miR‐708 sequence matching element on the *Dab2* 3ʹ UTR and the designed mutation version. (G) The maps of luciferase‐expressing reporter plasmids. pF‐CTL: without *Dab2* 3ʹ UTR; pF‐wDab2: with *Dab2* 3ʹ UTR containing wild type miR‐708 matching element; pF‐mDab2: with *Dab2* 3ʹUTR containing mutated miR‐708 matching element. (H) Luciferase activity in HEK293 cells transfected by indicated reporter plasmids with miR‐708 mimics or miR‐CTL. Relative folds of luciferase activity were shown. (I–N) NIH3T3 cells were transfected with miR‐708 mimics (miR‐708) alone or plus *Dab2* expression plasmid (DAB2), or with miR‐708 inhibitor (anti‐miR‐708) alone or plus siRNA for the *Dab2* gene (si‐DAB2), then cells receivedH_2_O_2_‐senescence induction treatment. (I and M): Representative images are shown by immunoblotting assays against DAB2 and p16 proteins. (K and L) Relative mRNA level of *Il‐6* and *Il‐8*. (J and N) Representative images are shown SA‐β‐gal staining (left) and average percentages of SA‐β‐gal positive cells (right). Scale bar represents 50 µm. All experiments repeated at least three times. n.s. *p* > 0.05, *p < 0.05, **p < 0.01, ***p < 0.001.

Progressing further, we analyzed the molecular pathway that drives the action of miR‐708 for aging protection. As shown in Figure 3A, we approached to identify target genes of miR‐708 through four steps. The first step was to predict and screen target genes having a miR‐708 binding site (s) on their 3′UTR by utilizing miRNA target prediction databases. The second step was to select the target genes, by pinpointing the intersection of predicted target genes with genes highly expressed in senescent cells, which have been profiled in our transcriptome data sheet previously.^29^ The third step was another round of selection, focusing on the target genes associated with metabolic regulation and stress response. Finally, we picked up gene called *Dab2*, because it encodes a protein participating in the signaling pathways likely to age progression, such as inflammation, immunity, differentiation, and cellular homeostasis.^30^, ^31^, ^32^ Our experimental data confirmed that the protein level of DAB2 was elevated in senescent cells (Figure 3B), and the mRNA level of the *Dab2* increased in the liver of D‐gal mice and the brain of naturally aged mice (Figures 3C and D). These findings suggest that *Dab2* is a possible miR‐708 target gene.

Further experiments the direct interplay between miR‐708 and the *Dab2* gene. One experiment ensured the influence of miR‐708 expression on *Dab2* expression. miR‐708 overexpression lowered DAB2 protein level in cells (Figure 3E). Another experiment affirmed the physical interaction and functional association between miR‐708 and the *Dab2* gene. As shown in Figure 3F, there was an element on the 3′UTR of the *Dab2* gene which completely matched with the core sequence of miR‐708. So that, luciferase expressing reporter genes with wild type or mutant 3′UTR of the *Dab2* gene (pF‐wDab2 or pF‐mDab2) were constructed (Figure 3G). By cotransfecting these reporter genes and with miR‐708 mimics, it turned out that miR‐708 overexpression only reduced the luciferase activity in wild tyle reporter transfected cells, but not in mutant reporter transfected cells (Figure 3H). These results demonstrate that miR‐708 functionally targets the *Dab2* gene by interacting with a specific binding site on the gene's 3UTR.

Then, we tested the correlation of miR‐708‐mediated DAB2 downregulation with the role of miR‐708 in senescence protection. Results showed that (1) the extent of endogenic DAB2 expression correlated with p16 protein abundance, SA‐β‐gal staining level, and SASP factors expression in cells (Figures 3I–K); (2) the ectogenic overexpression of DAB2 vanished miR‐708 mimics‐induced reductions in these senescence indicators (Figures 3I–K); (3) siRNA‐DAB2 vanished miR‐708 inhibitor‐induced increments in these senescence indicators (Figures 3L–N). These results suggest that the DAB2 works downstream of miR‐708 and plays negatively for the role of miR‐708 in protection from senescence.

### 2.5 MiR‐708 withstands senescence through suppressing DAB2/mTOR signaling

**FIGURE 4:**
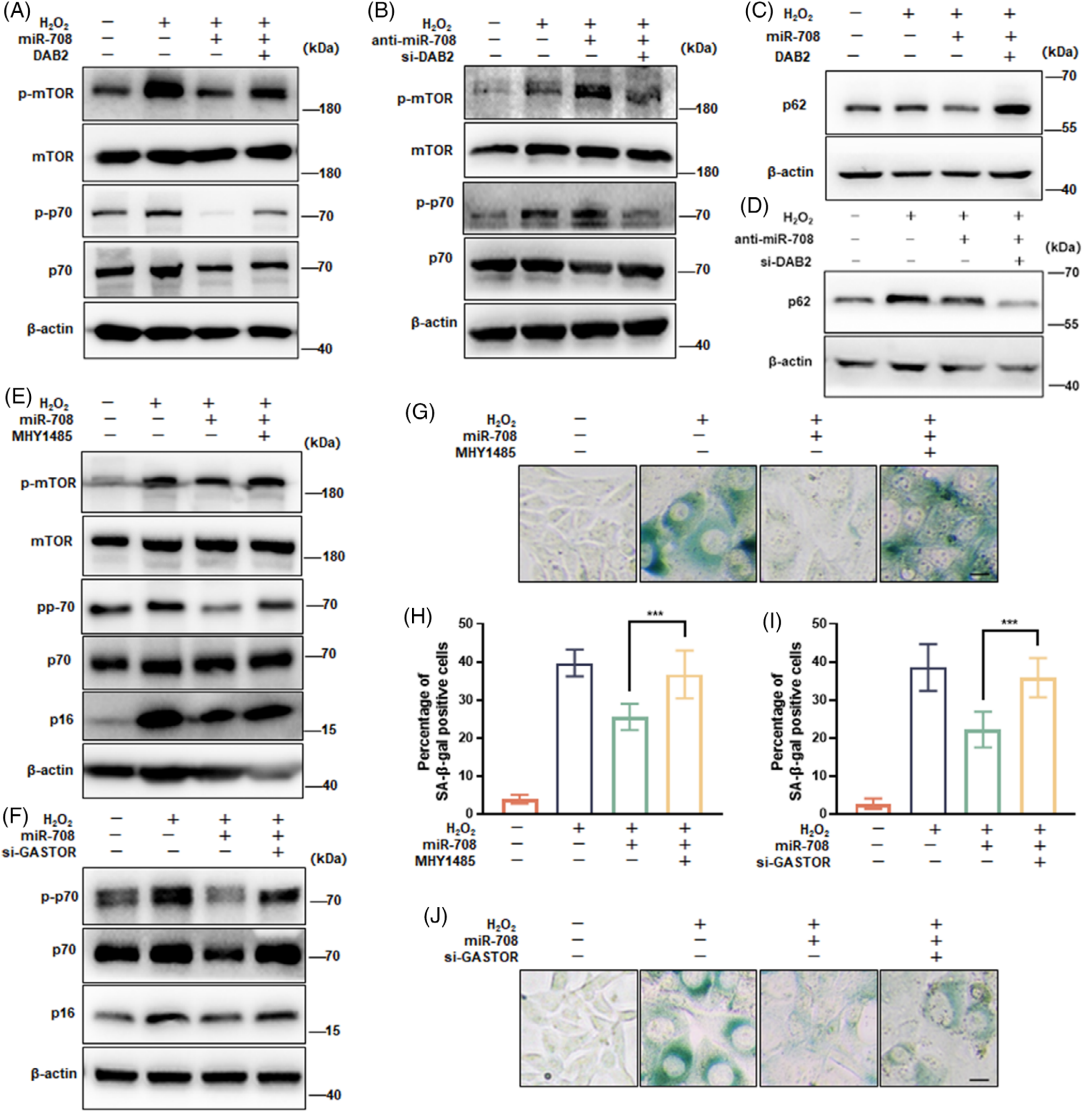
miR‐708 prevents cellular senescence by suppressing DAB2/mTOR pathway. (A–D) NIH3T3 cells were transfected with miR‐708 mimics (miR‐708) alone or plus DAB2 expression plasmid (DAB2), or with antisense miR‐708 (anti‐miR‐708) alone or plus siRNA for the *Dab2* gene (si‐DAB2), then cells received H_2_O_2_‐senescence induction treatment. (E, G, and H) miR‐708 alone transfected cells a received H_2_O_2_‐senescence induction treatment with or without MHY1485. (F, I, and J) miR‐708 alone or plus siRNA against GASTOR (si‐GASTOR) transfected cells received H_2_O_2_‐senescence induction treatment. (A–F) Representative images are shown by immunoblotting assays against indicated proteins. (G and J) Representative images are shown SA‐β‐gal staining. (H and I) Average percentages of SA‐β‐gal positive cells. Scale bar represents 50 µm. The statistical data were the mean values of three independent experiments. ***p < 0.001.

As DAB2 is an adaptor protein interplaying with the AKT–mTOR pathway and the known association of this pathway with aging regulation,^33^, ^34^ we next investigated the relationship of miR‐708 and DAB2 with mTOR activity and the implication of this relationship for senescence occurrence. As shown in Figure 4, both phosphorylations of mTOR and p70/S6K were elevated in senescent cells, signing the status of the aberrant activation of mTOR. Importantly, these elevated phosphorylations were blunted when cells with miR‐708 overexpression, but persisted when cells with miR‐708 overexpression plus DAB2 overexpression (Figure 4A). In contrast, these elevated phosphorylations got strengthened by miR‐708 inhibition, not by miR‐708 inhibition with DAB2 silence (Figure 4B). We also measured the cellular autophagy status due to it is negatively regulated by mTORC1and positively correlated with aging prevention. As shown in Figures 4C and D, miR‐708 overexpression remarkably relieved the aberrant accumulation of p62 protein in senescent cells, a well‐accepted indication of autophagy impairment, but this relief was vanished, and when DAB2 silenced. The negative implication of mTORC1 on the antiaging effect of miR‐708 was further collected. As shown, either MHY1485, a chemical activator of mTORC1, or siRNA against GASTOR, an inhibitory protein of mTORC1, erased the decline of p16 protein abundance in senescent cells caused by miR‐708 overexpression (Figures 4E and F). Likewise, MHY1485 and si‐GASTOR blunted the miR‐708 overexpression‐caused decline of SA‐β‐gal staining (Figures 4G–J). Altogether, these results indicate that the suppression of DAB2 expression and mTOR activity are of importance for the role of miR‐708 in senescence protection.

### 2.6 MiR‐708 expression is positively regulated by AMPK

**FIGURE 5:**
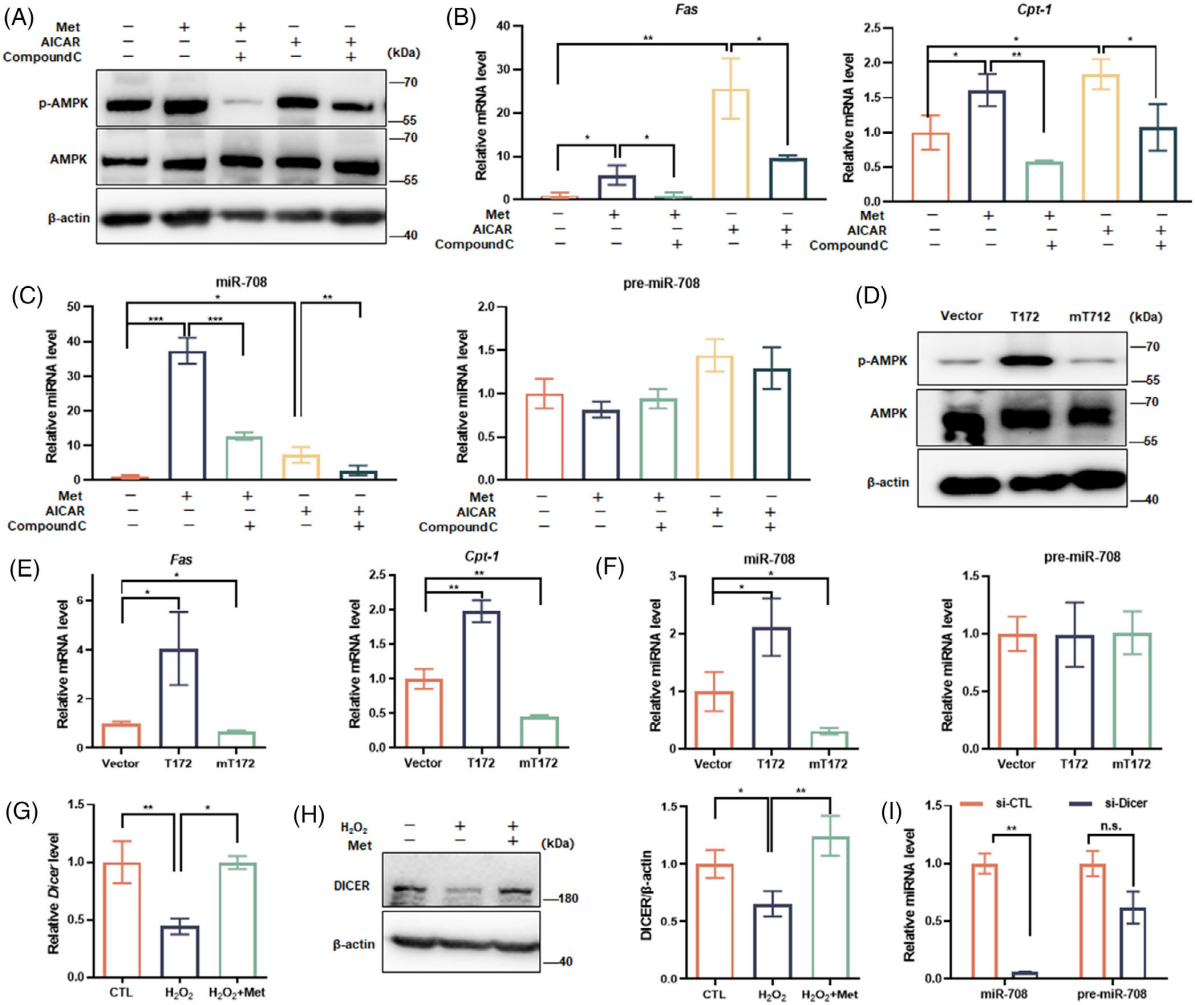
AMPK upregulates miR‐708 expression in a posttranscriptional level. (A–C) NIH3T3 cells received H_2_O_2_‐senescence induction with or without Met, AICAR or Compound C addition. (D–F) The expression of wild type AMPK (T172) or inactive mutated AMPK (mT172) and control (Vector) NIH3T3 cells were treated with H_2_O_2_‐senescence induction procedure. (A and D) Representative images are shown by immunoblotting assays against indicated proteins. (B and E) Relative mRNA levels of *FAS* and *CPT‐1* genes were evaluated. (C and F) Relative levels of maturated miR‐708 and pre‐miR‐708 were assessed. (G and H) Cells received H_2_O_2_‐senescence induction treatment with or without Met. (G) Relative mRNA level of *Dicer*. (H) Representative images are shown by immunoblotting assays (left) and the relative level of DICER protein normalized by β‐actin level were shown (right). (I) Cells were transfected with scrambled siRNA (si‐CTL) or siRNA for the *Dicer* gene (si‐Dicer) for 24 h, followed by H_2_O_2_ and Met treatments, levels of maturated miR‐708 and pre‐miR708 were assessed. All experiments were repeated at least three times. n.s. *p* > 0.05, *p < 0.05, **p < 0.01, ***p < 0.001.

In addition to the downstream signaling of miR‐708, the upstream signaling of miR‐708 was also addressed. As shown in Figure 1C, miR‐708 expression not only decreased in senescent cells, but also increased in metformin‐treated cells, consistent with the finding that metformin can upregulate the expression of miR‐708 in human breast cancer cells.^35^ Considering that metformin is an AMPK activator, we set about to evaluate the relationship between AMPK and miR‐708 expression. Both pharmacological and genetic approaches were applied. As the result, AICAR, a direct agonist of AMPK, and Compound C, a direct inhibitor of AMPK, clearly altered the expression of miR‐708 (Figure 5C), similarly as they did for the phosphorylation of AMPK (Figure 5A) and the transcription of the *Fas* gene and the *Cpt‐1* gene, both accept the positive regulation of AMPK^36^ (Figure 5B). Accordingly, wild type AMPK overexpression resulted in the increment of miR‐708, while the inactive mutant AMPK overexpression did not (Figures 5D–F). These results ascertain that AMPK is a positive regulator of miR‐708 expression.

Intriguingly, AMPK activation only upregulated the mature form of miR‐708, named as miR‐708, but not its precursor, pre‐miR‐708 in our study (Figures 5C and F). It enlightened us to play the particular attention on the posttranscriptional regulation of AMPK on miR‐708 expression. Grounding on the information that DICER, a master enzyme governing the processing program from pre‐miRNA to mature miRNA, was downregulated during aging and AMPK activation could upregulate its expression (Figures 5G and H), we thought of the possibility that AMPK‐upregulated miR‐708 expression may depend, at least in part, on AMPK‐mediated DICER expression. As expected, silencing *Dicer* by siRNA approach lowered the level of AMPK‐mediated miR‐708 upregulation, while not the level of pre‐miR‐708 in senescent cells (Figure 5I). These results demonstrate that miRNA maturation is crucial step for the influence of AMPK on miR‐708 expression, in which AMPK elevates DICER expression and DICER promotes miR‐708 maturation.

### 2.7 MiR‐708 mediates the anti‐aging effect of AMPK

**FIGURE 6:**
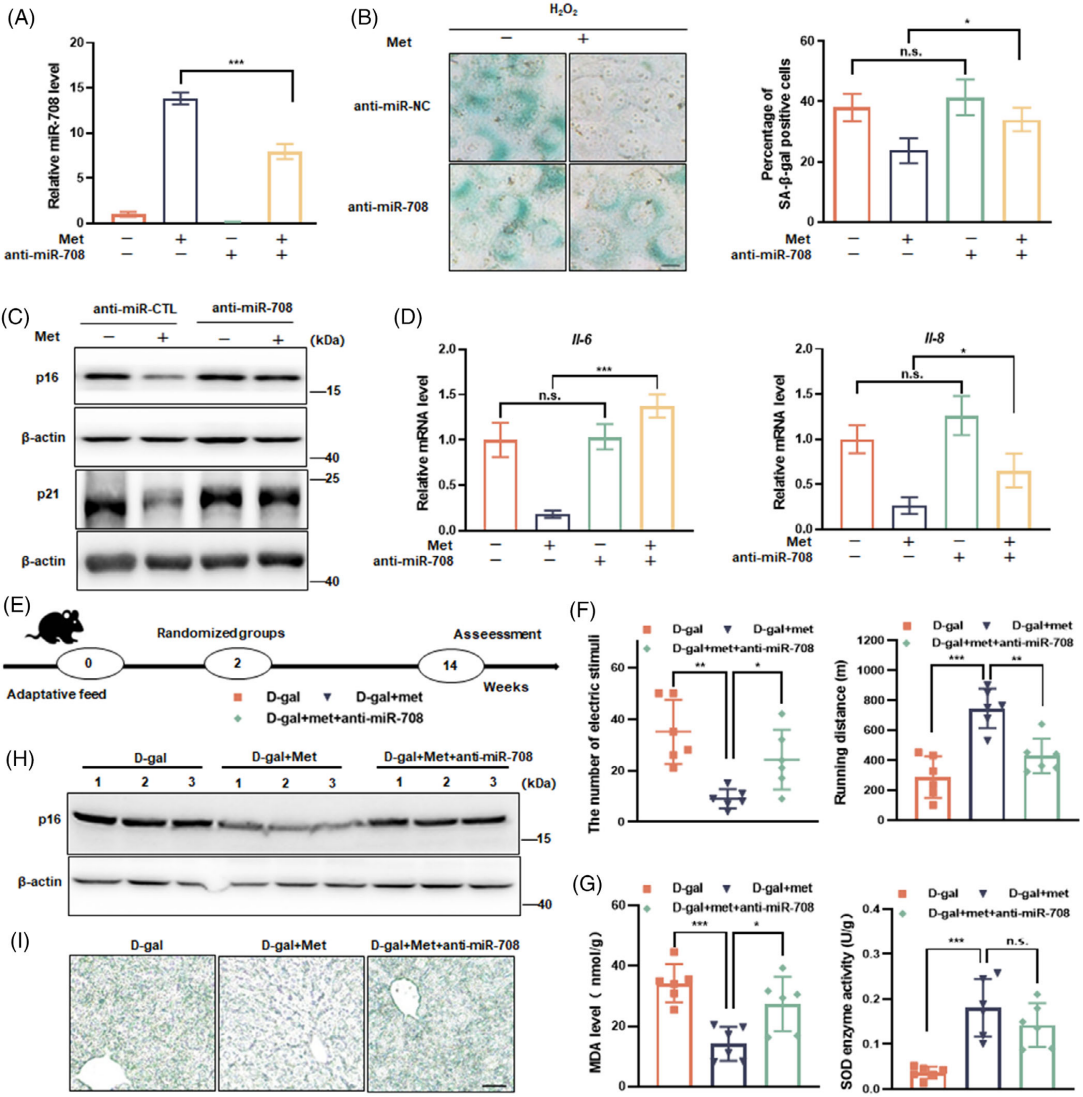
miR‐708 participates in the anti‐aging effect of AMPK. (A–D) NIH3T3 cells were transfected with antisense miR‐708 (anti‐miR‐708) or scrambled miRNA (anti‐miR‐CTL) for 24 h, followed by the H_2_O_2–_induced senescence with or without Met intervention. (A) Relative miR‐708 level. (B) Representative images are shown SA‐β‐gal staining (left), and average percentage of SA‐β‐gal positive cells based on three independent experiments (right). (C) Representative images are shown by immunoblotting assays against indicated p16 and p21. (D) Relative mRNA level of *Il‐6* and *Il‐8*. (E) The experimental procedure (*n* = 6) (F) Electric stimuli times (left) and running distance (right), recorded by treadmill. (G) MDA level (left) and SOD activity (right) in liver. (H) Representative images are shown by immunoblotting assays against indicated p16 in liver. (I) Representative images of SA‐β‐gal staining of liver sections. Scale bar indicates 50 µm. All cell culture experiments were repeated at least three times. n.s. *p* > 0.05, *p < 0.05, **p < 0.01, ***p < 0.001.

Further experiments were performed for affirming the functional implication of miR‐708 on the antiaging effect of AMPK. In cell culture experiments, the transfection of miR‐708 inhibitor (anti‐miR‐708) significantly weakened the suppressive effect of metformin on SA‐β‐gal staining, p16 and p21 expression, and also the expression of SASP factors (Figures 6A–D). To get in vivo evidence, the AAV‐mediated intervention in mice was engaged (Figure 6E). As shown, the transduction of AAV9‐anti‐miR‐708‐mCherry (anti‐miR‐708) got succeed as strong fluorescence accumulated in mouse liver, consistently with the molecular assay data showing this transduction effectively suppressed miR‐708 expression (Figure S5A). Importantly, this transduction counteracted the upregulation effect of metformin on miR‐708 expression (Figure S5B). As for aging‐related parameters, anti‐miR‐708 blunted the restoring effects of metformin on physical strength and memory ability of mice (Figures 6F and S5C and D), and also weakened the improving effects of metformin on ALTL activity and MDA generation, although no influence for the effect of metformin on ASTL activity and SOD activity (Figures 6G and S5E). Finally, AAV9‐anti‐miR‐708‐mCherry transduction impaired the suppressive effects of metformin on the p16 expression and the SA‐β‐gal staining in liver of aging‐like mice (Figures 6H and I). These findings reveal that miR‐708 acts downstream of AMPK and plays as a part for the antiaging effect of AMPK in mice.

## 3 DISCUSSION

In combination with differential expression detection, bioinformatic analysis, and experimental investigation, we identified a new SA‐miRNA, miR‐708, in this study and analyzed its mechanism. We found: (1) miR‐708 is an antiaging miRNA; (2) the antiaging mechanism of miR‐708 is associated with its direct targeting of DAB2, that then inhibits mTOR activity and improves autophagic activity; (3) miR‐708 expression is positively regulated by AMPK activity, depending on AMPK‐elevated DICER expression and DICER‐driven miR‐708 maturation. These findings propose for the first time that miR‐708 is an antiaging SA‐miRNA and displays originally that this role of miR‐708 is dependent on a cascade that connects mTORC1 and AMPK together, in which miR‐708 suppressed DAB2 expression acts as a key step that passes the negative implication of AMPK on mTORC1. We hope these distinct findings could inspire further investigation for aging regulation mechanism and the biomedical value of miR‐708.

Aging is a complex biological process, progressing via various molecular mechanisms, such as cell cycle arrest, telomere shortening, inflammatory process, metabolic disorder, and organelle dysfunction.^37^ All these mechanisms affect gene expression, regulating at the transcriptional level and/or posttranscriptional level. Many studies explored transcriptional regulation mechanisms for aging‐related genes, such as the p16 gene and inflammatory genes.^38^, ^39^ Posttranscriptional regulation mechanisms of aging‐related genes are known relatively less but attract increasing attention in recent years, partially owing to the improved understanding of the biological role of miRNAs. Up to now, several dozens of aging‐related miRNAs have been identified, generally named as SA‐miRNAs. From a functional point of view, the most identified SA‐miRNAs act for aging promotion, by targeting genes involved in the regulation of cell cycle, inflammatory response and telomere replication.^11^, ^12^, ^40^ That is different from miR‐708 we identified in this study.

The most of knowledge for miR‐708 is about its involvement in tumorigenesis and immune response.^41^, ^42^ But a few studies indicated the alteration of miR‐708 expression in aging. For example, Noren et al.^23^ showed that miR‐708 expression reduced in blood of the elderly; Lee et al.^24^ reported that miR‐708 expression positively associated with longer lifespan of mice. Consistent with these clues, the aging protective role of miR‐708 got cleared by in the present study, with the causal relationship verification based on in vitro and in vivo experiments. Although experimental data from naturally aged mice should be more ideal, our results from D‐gal‐injected mice are still valuable since plenty of investigations have revealed the presenile phenotypes, molecular and pathophysiologic aging alterations of this model mice, such as decreases in physical power and cognition ability, elevated signs of oxidative stress and DNA damage, higher expression of p16 and proinflammation factors in tissues, and obvious degenerative alterations in organs.^25^, ^26^, ^27^

Our finding that miR‐708 can directly target DAB2 is intriguing and interesting. Although DAB2 expression can be regulated by other miRNAs, like miR‐93 and miR‐191,^43^, ^44^ its relationship with SA‐miRNAs has not been reported before. In a matter of speaking, several pieces of information are supportive for our finding, which demonstrated the implication of DAB2 in AKT–mTOR pathway regulation,^33^ or the aging‐promoting implication of consistent mTOR activation.^45^, ^46^ Moreover, the association between DAB2 and autophagy has been reported in previous. The relevant stated that DAB2 can obstruct the formation of the BECLIN‐1–VPS34 complex and subsequently inhibit autophagy processes.^30^ In the present study, we explored another mechanism to explain the autophagy‐inhibitory role of DAB2, that is the mTORC1 pathway activation. It is worth to mention that the finding based on this study seems contradictory to some other studies, which demonstrated that DAB2 acts for AKT inactivation and protein synthesis suppression,^33^, ^47^ whereas in our study context, DAB2 worked positively for senescent‐associated mTOR activation. We could not explain this discrepancy confidently up to now, just keep two possibilities in mind for further consideration. One possibility is that DAB2 may work with a protein complex different from complex AKT involved, and this complex affects positively mTOR activity in the senescence context. Another possibility is that the effect of DAB2‐mediated AKT inactivation on mTORC1 activity may become reprogrammed in a context where mTORC1 is sustainedly activated, like in senescent cells, where a novel reciprocal relationship established between AKT–mTORC1 cascade and mTORC2‐AKT cascade.^48^ Anyway, we believe that either the relationship between miR‐708 and DAB2, or the association between DAB2 and mTORC1, is not one‐to‐one specific, so that other pathways and mechanisms could not be neglected when discussing about the aging‐protective role of miR‐708 and the aging‐promoting role of DAB2.

AMPK‐prompted miR‐708 maturation is also an intriguing finding of this study. It was first hinted in our miRNA differential expression profile, and then ensured by comparing the effect of metformin on pre miR‐708 expression and maturated miR‐708 expression. Like the majority of other miRNAs, miR‐708 processing goes through the formations of pri‐miR‐708, pre‐miR‐708, and mature miR‐708 in succession, where the stage for the transition from pre‐miR‐708 to miR‐708 are managed by DICER and AGO enzymes.^49^ Consistent with the information that AMPK activity can increase the expression of DICER and both of them declined with aging,^50^, ^51^ our investigation verified that AMPK can upregulate DICER expression and DICER is necessary for miR‐708 expression. In fact, these findings are consistent with those proposed by others, even though previous studies are not executed in the aging‐ or senescence‐relevant system. For instance, Tan et al.^35^ found that miR‐708 expression was significantly elevated in metformin‐treated human breast cancer cell lines in a dose‐dependent manner. From this point of view, miR‐708 is an important mediator for the antiaging effect of AMPK.

In conclusion, this study demonstrates that miR‐708 poses antiaging property, and acts at least in part by the direct inhibition of DAB2 expression. It also proposes a novel signaling cascade where AMPK and miR‐708 at upstream, and DAB2 and mTORC1 at downstream, and reveals a mechanism by which AMPK promotes miR‐708 maturation via upregulating DICER, and miR‐708 suppresses DAB2 expression that consequently blocks the aberrant mTOR activation, so that to prevent cellular senescence and aging. This finding has promising significance for further studies, because it identified a new antiaging molecule miR‐708 and figured out a new but targetable cascade for aging regulation. Not only, the elucidated miR‐708 action and regulation expand our understanding about the aging protection mechanism of AMPK. Evidence needs to be collected to solidly verify the molecular cascade flux we proposed, particular those sampling from primate and human body.

## 4 MATERIALS AND METHODS

### 4.1 Cell culture and treatments

NIH3T3, MRC‐5 and HEK293 were purchased from Shanghai Institutes for Biological Sciences of Chinese Academy of Sciences (Shanghai, China); human primary fibroblasts cells (HFs) were obtained from the human primary sphenoid sinus mucosa tissues.

H_2_O_2_‐induced cellular senescence: NIH3T3 and MRC‐5 cells were treated with H_2_O_2_ (400 or 100 µM, respectively) for 1 h and then incubated with complete medium for 3 days as described previously.^18^, ^19^ ADR‐induced cellular senescence: NIH3T3 cells were treated with 1 µM of ADR for 6 days, with once medium change on the third day. Replicative senescence: HFs cells were cultured routinely and split 1:3 for passaging until they reached apparent growth arrest with phenotypes of senescence as previously described.^52^ Cells were incubated with Met (8 mM), AICAR (10 µM), Compound C (10 µM), or MHY1485 (10 nM) in senescence process.

### 4.2 Acquisition and analysis of miRNA expression profile

H_2_O_2_‐induced senescent cells, Met‐intervened senescent cells and their homologous proliferating cells (NIH3T3) were cultured and collected for small RNA sequencing. Nine samples from three dependent experiments were sequenced by Novogene (Beijing, China) and analyzed by Chengdu Bayer Biotechnology Corporation (Chengdu, China). DESeq analysis was used to identify differentially expressed miRNAs with a threshold of fold change ≥1.5 and *p *< 0.01.

### 4.3 miRNA and siRNA transfection

Cells were transfected with miRNA/siRNA (50 nm) using transfection reagent (jetPRIME; Polyplus, France) for 6−8 h, then replaced with fresh DMEM medium. The miRNA and siRNA preparations were synthesized by GenePharma (Shanghai, China) with following sequences: mmu‐miR‐CTL: CAGUACUUUUUGUGUAGUACAA, mmu‐miR‐708 mimics, the synthetic RNA fragment with the sense sequence of miR‐708 (miR‐708): AAGGAGCUUACAAUCUAGCUGGG, mmu‐miR‐708 inhibitor, the synthetic RNA fragment with the antisense sequence of miR‐708 (anti‐miR‐708): CCCAGCUAGAUUGUAAGCUCCUU, pre‐miRNA: CUGUGUUUUGAAAUGGGGACUGCCCUCAAGGAGCUACAAUCUAGCUGGGGGUAGAUGACUUGCACUUGAACACAACUAGACUGUGAGCUUCUAGAGAGGGCAGGGGCCUUA, mmu‐si‐CTL positive strand: ’'‐UUCUCCGAACGUGUCACGUTT‐’', antisense strand ’'‐ACGUGACACACGUUCGGAGAATT‐’', mmu‐si‐Dab2 positive strand: ’'‐GAGTCAATGTCCAAATACT‐’', antisense strand ’'‐AGTATTTGGACATTGACTC‐ ’'.

### 4.4 Quantitative real‐time PCR

RNA samples were collected using Trizol reagent (Sangon Biotech, Shanghai, China) following the manufacturer provided protocol. For cDNA synthesis or miRNA RT–PCR, reverse transcription was performed using RT SuperMix (R123‐01; Vazyme, Nanjing, China) or All‐in‐One™ miRNA First‐Strand cDNA Synthesis Kit (QP114; GeneCopeia, Guangzhou, China), respectively, according to manufacturer's protocols. Real‐time PCR was conducted using the 2* SYBR Green qPCR Master Mix (B21202; Bimake) on the Real‐Time System (ABI, USA). Primer sequences are listed in Table S1.

### 4.5 Western blot

Collecting, blotting, imaging, and quantification of protein samples were conducted using the standard reagents and protocols. The following antibodies and dilutions were used: β‐actin (bs‐10966R; Bioss Antibodies; 1:1000), p16 (ab51243; Abcam; 1:1000), p21 (2947S; CST; 1:1000),DAB2 (12906S; CST; 1:500), p70 (A5512; Bimake; 1:1000), p‐p70 (9204S; CST; 1:1000), mTOR (2983S; CST; 1:1000), p‐mTOR (5536S; CST; 1:1000), p62 (ab109012; Abcam; 1:2000), AMPKα (5831S; CST; 1:1000), p‐AMPKα (2535S; CST; 1:1000), DICER (ab227518; Abcam; 1:500), goat anti‐Rabbit IgG (H&L) HRP conjugate (701051; ZEN‐BIOSCIENCE; 1:10000).

### 4.6 SA‐β‐gal staining

SA‐β‐gal activity in cells was measured detected in situ using β‐galactosidase staining kit (G1580; Solarbio, Beijing, China) according to manufacturer's protocol. The percentage of SA‐β‐gal positive cells with blue‐green coloring were calculated based on the images from a phase contrast microscopy. Five random fields of microscope were selected for counting in each group, and the experiment was repeated at least three times independently.

### 4.7 Human blood sample acquisition and detection

The study was approved by the Medical Ethics Committee of The Second People's Hospital of Changzhi City. Human venous blood was drawn in the morning after 12 h fasting and centrifuged at 600 g for 10 min and discard the supernatant. Precipitation is fully lysed by red blood cell lysate (B541001; Sangon Biotech), and centrifuged at 900 *g* for 10 min. Separated specimens were stored in a −80°C refrigerator or performed subsequent miRNA extraction and detection.

### 4.8 Animal experiments

6−8 week‐aged male C57BL/6J mice were purchased from the HuaFuKang Biotechnology Company (Beijing, China). Mice were kept in an SPF‐grade environment with free access to feed and water.

D‐gal‐induced accelerated aging model were performed as described previously.^25^, ^26^, ^27^ After 2 weeks of adaptive feeding, mice were randomly divided into five groups (*n* = 6): the control group (saline group), the aging‐like model group (D‐gal group), miR‐708 intervention group (D‐gal+miR‐708 group), Met intervention group (D‐gal+Met group), and miR‐708 and Met cointervention group (D gal+Met+anti‐miR‐708 group). Saline group mice were given saline only, D‐gal group mice accepted daily 125 mg/kg D‐gal injection (i.p.) for 3 months, and D‐gal+miR‐708 group mice accepted D‐gal injection, together with one‐time injection of miR‐708/GFP‐AAV9 (AA09‐MmiR3115‐MR14‐400; GeneCopoeia, Guangzhou, China) at the beginning of the experiment (2 × 10^11^ GC), D‐gal+Met group mice accepted D‐gal injection also, together with Met (100 mg/kg/day), and D‐gal+Met+anti‐miR‐708 group mice accepted D‐gal and Met injection together with anti‐miR‐708/mcherry‐AAV9. D‐gal was purchased from Sigma–Aldrich (St. Louis, MO, USA); Met was purchased from MUSTBIO technology (Chengdu, China); AAV9 carrying miR‐708 and anti‐miR‐708 were constructed and packaged by GeneCopoeia (AA09‐MmiR‐AN0783‐AM07‐400). Aging‐related indices were evaluated after 3 months and after mice were sacrificed. After the gavage, the blood, liver, brain, and kidney tissues were isolated completely and collected for subsequent experiments.

### 4.9 The ethological experiments

(1) Endurance test: mice were put on a mouse treadmill (Ugo Basile, Milan, Italy), that was set at an incline of 5%, running speed of 15 m/min, running time of 1 h, electrical stimuli (current intensity of 1 mA and the threshold of 50 shocks). As running practice, mice were accustomed to run on the treadmill 30 min/day for 3 consecutive days before the experiment. The endurance of mice was assessed based on the running distance and the number of electrical stimuli.
(2) Grip strength test: a grip strength meter (Saiangsi, Jiangsu, China) was used to assess the grip strength of mice forelimbs. When the griping net was attached to the forelimb of mice, the tail of each mouse was pulled back gently and in parallel, and the tension reading was defined as the forelimb grip strength (*g*) before net was released by mouse. Each mouse accepted three consecutive tests and the mean of grip strengths was taken.
(3) Memory test: the short memory of mice was evaluated using a Y‐maze apparatus (Saiangsi). The experiment was divided into training phase and formal phase. In training phase: the novel arm was closed, and each mouse was put into the start arm, who are allowed to move freely in the start arm and the other arm for 10 min. In formal phase: After 1 h of training, the novel arm was opened, and each mouse put into the start arm moved freely in all three arms and its trace were recorded for 5 min. The memory of each mouse was calculated as a score of spontaneous alteration = total number of alterations/total number of arm entries × 100%.

### 4.10 MDA level and SOD activity measurement

MDA level or SOD activity in liver was measured using an MDA content detection kit (BC0025; Solarbio) or SOD activity detection kit (BC0175; Solarbio) following the manufacturer's protocol.

### 4.11 Lentivirus construction and cell‐line screening

The coding region fragment of *Dab2* was obtained by amplifying mouse cDNA with Phanta® Super‐Fidelity DNA Polymerase (P511‐01; Vazyme), then inserted into the pLVX‐Puro lentiviral expression vector (Clontech, Shiga, Japan). miR‐708 and miR‐CTL plasmids were purchased from GeneCopoeia (Guangzhou, China). The lentivirus of *Dab2*, miR‐708 and miR‐CTL were separately packaged, transduced, and screened according to standard protocol.

### 4.12 Dual‐luciferase reporter assay

Two special luciferase expressing reporters were established, carrying the wide type 3′UTR of the *Dab2* gene (pF‐wDab2), and the mutated 3′UTR of the *Dab2* gene (pF‐mDab2), respectively. The mutated one lost the miR‐708 binding site, and an empty reporter without the 3′UTR of the *Dab2* gene was used as the negative control (pF‐CTL). These reporters were cotransfected with miR‐708 mimics or miR‐CTL into HEK293 cells using the transfection reagent (jetPRIME; Polyplus). After 48 h, the cells were collected and the luciferase activities were measured sing a dual‐luciferase reporter assay (Vazyme; DD1205‐01) according to manufacturer's protocol. Three independent experiments were carried out.

### 4.13 Statistical analysis

The statistical data were represented as the mean ± SD from at least three independent experiments. GraphPad Prism 8 software were used to assess statistical significance. Comparisons between two groups were performed using an unpaired Student's *t*‐test. Differences were analyzed one‐way ANOVA followed by Dun's test for multiple comparisons. Statistical significance was set at *p *< 0.05.

## AUTHOR CONTRIBUTIONS

Jian Zhang designed and supervised the study, carried out most of the experiments, analyzed the data, prepared the figures, and wrote the draft of the manuscript. Hui Gong, Tiepeng Li, Ming Yang, Chuhui Gong, Ning Huang, Yu Yang, and Cuiying Zhang performed some experiments. Tingting Zhao, Weitong Xu, Honghan Chen, Fangfang Wang, and Jin Liu contributed to data analysis and manuscript preparation. Hengyi Xiao conceived and designed the concept of this study, and revised this manuscript. All authors reviewed and approved the final version of the manuscript.

## CONFLICT OF INTEREST STATEMENT

The authors declare that they have no conflict of interest.

## ETHICS STATEMENT

In this study, the collection of clinical samples has obtained the approval of the Medical Ethics Committee of The Second People's Hospital of Changzhi City (CZEYLI‐007). All animal experiments were conducted in accordance with protocols approved by the Laboratory Animal Ethics Committee of West China Hospital, Sichuan University (IACUC, 2022081204), and all operating procedures followed the guide for the Care and Use of Laboratory Animals.

## Supporting information

Supporting Information

## Data Availability

The small‐RNA sequencing data included in this study are available in the NCBI Sequence Read Archive (SRA). The SRA under the following accession numbers: SRR10828466, SRR10828465, SRR10828464, SRR10828463, SRR10828462, SRR10828461, SRR10828460, SRR10828459, and SRR10828458. Other data in this study are available from the corresponding author upon reasonable request.
