# Correction to “AMPK‐upregulated microRNA‐708 plays as a suppressor of cellular senescence and aging via downregulating disabled‐2 and mTORC1 activation”

**DOI:** 10.1002/mco2.70526

**Published:** 2025-12-08

J. Zhang, H. Gong, T. Zhao, et al., “AMPK‐upregulated microRNA‐708 plays as a suppressor of cellular senescence and aging via downregulating disabled‐2 and mTORC1 activation,” *MedComm (2020)* **5**, no. 3 (2024): e475.

In paragraph 7 of the “Acknowledgements” section, the text “National Natural Science Foundation of China (Grant No. 82101629 to Hui Gong), Post‐Doctor Research Project, West China Hospital, Sichuan University (Grant No. 2021HXBH003 to Hui Gong)” was incorrect.

This should have read: “National Natural Science Foundation of China (Grant Nos. **82101629** to Hui Gong), **Sichuan Science and Technology Program (**Grant No. **2023NSFSC1236** to Hui Gong), Post‐Doctor Research Project, West China Hospital, Sichuan University (Grant No. **2021HXBH003** to Hui Gong).”

We apologize for this error.

