# Correction to “AMPK‐upregulated microRNA‐708 Plays as a Suppressor of Cellular Senescence and Aging via Downregulating Disabled‐2 and mTORC1 Activation”

**DOI:** 10.1002/mco2.71077

**Published:** 2026-10-13

J. Zhang, H. Gong, T. Zhao, et al., “AMPK‐Upregulated Microrna‐708 Plays as a Suppressor of Cellular Senescence and Aging via Downregulating Disabled‐2 and mTORC1 Activation,” *MedComm* 5, no. 3 (2024): e475, https://doi.org/10.1002/mco2.475.

In Figure 1G of the “Results” section, the quantitative real‐time PCR graph was inadvertently duplicated from Figure 1F. The corrected Figure 1G is shown below.


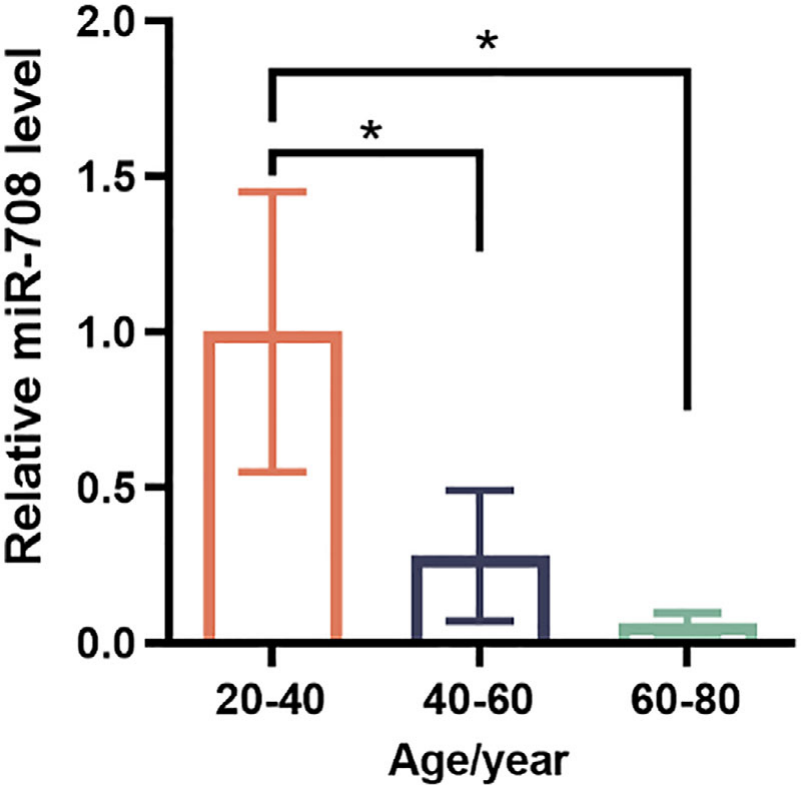


In the “Materials and Methods” section, subsection “4.5 Western blot”, the antibody information stated as “p16 (ab51243; Abcam; 1:1000)” was incorrect. The correct information is “p16 (A0262; ABclonal; 1:1000)”.

These corrections do not affect the scientific results or conclusions of the article.

We apologize for these errors.

