# Supplementary material for: AMPK‐upregulated microRNA‐708 plays as a suppressor of cellular senescence and aging via downregulating disabled‐2 and mTORC1 activation
Source: MedComm (2020). 2024 Mar 9;5(3):e475. doi: 10.1002/mco2.475 (PMC10924637; doi:10.1002/mco2.475)
Supplement: Supplementary file 1 — Supporting Information [file MCO2-5-e475-s001.pdf]

AMPK-upregulated microRNA-708 plays as a suppressor of cellular senescence and aging via downregulating Disabled-2 (DAB2) and mTORC1 activation

Running title: AMPK suppresses aging via miR-708/DAB2/mTOR axis

Jian Zhang[1, 2], Hui Gong[1], Tingting Zhao[1], Weitong Xu[1], Honghan Chen[1], Tiepeng Li[1], Yu Yang[1], Ming Yang[1], Ning Huang[1], Chuhui Gong[1], Fangfang Wang[1], Cuiying Zhang[3], Jin Liu[1], Hengyi Xiao[1]*

Affiliations:

1. The lab for Aging Research, National Clinical Research Center for Geriatrics, State Key Laboratory of Biotherapy, West China Hospital, Sichuan University, 1 Keyuan 4 Road, Gaopeng Avenue, Chengdu, 610041, China.

2. Department of Biochemistry and Molecular Biology, and Molecular Medicine and Cancer Research Center, College of Basic Medical Sciences, Chongqing Medical University, Chongqing, 400016, China

3. Departments of Laboratory Medicine, The Second People's Hospital of Changzhi City, Changzhi, 046000, China.

*** Corresponding author:** H. Xiao, The Lab for Aging Research, National Clinical Research Center for Geriatrics, State Key Laboratory of Biotherapy, West China

Hospital, Sichuan University, 1 Keyuan 4 Road, Gaopeng Avenue, Chengdu, 610041, China. E-mail: hengyix@scu.edu.cn

SUPPLEMENTARY TABLE 1. Primers sequences used for qPCR

Genes	Forward primer	Reverse primer
18S	TTGACGGAAGGGCACCACCAG	GCACCACCACCACGGAATCG
P21	GTGGCCTTGTCGCTGTCTT	GCGCTTGGAGTGATAGAAATCTG
P53	GTCACAGCACATGACGGAGG	TCTTCCAGATGCTCGGGATAC
Il-1α	CGAAGACTACAGTTCTGCCATT	GACGTTTCAGAGGTTCTCAGAG
Il-6	TAGTCCTTCCTACCCCAATTTCC	TTGGTCCTTAGCCACTCCTTC
Il-8	CAGCTGCCTTAACCCCATCA	CTTGAGAAGTCCATGGCGAAA
Dab2	CCTTCATTGCTCGTGATGTGA	CCCCAAACAAATCCATCTGGTC
Fas	GGAGGTGGTGATAGCCGGTAT	TGGGTAATCCATAGAGCCCAG
Cpt-1	CTCCGCCTGAGCCATGAAG	CACCAGTGATGATGCCATTCT
U6	CTCGCTTCGGCAGCACA	AACGCTTCACGAATTTGCGT
miR-708	AAGGAGCUUACAAUCUAGCUGGG	-

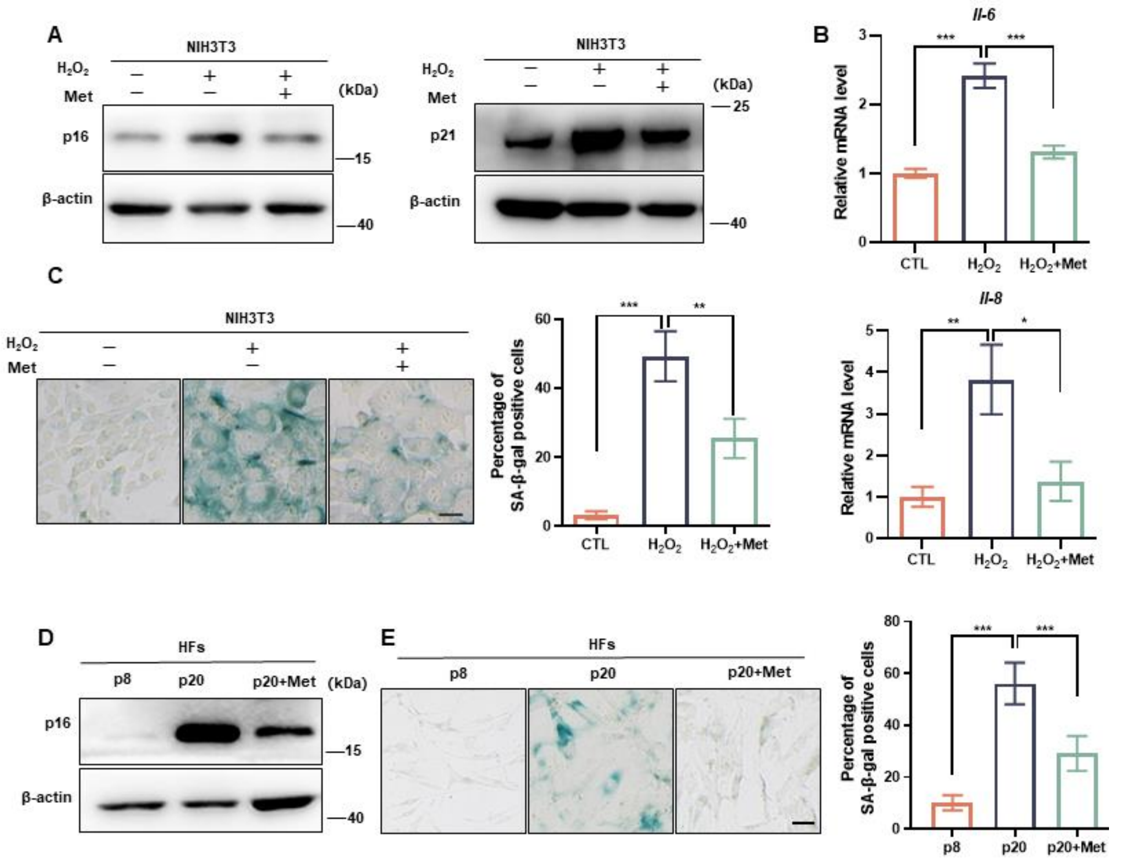

SUPPLEMENTARY FIGURE 1

AMPK alleviates cellular senescence. A-C: NIH3T3 cells were induced by the senescence induction. D, E: HFs of passage 8th were cultivated and continuously passaged with or without Met until passage 20th. Cells at passage 8th and passage 20th were harvested for assays. (A, D) Representative images are shown by immunoblotting assays against p16 and p21 proteins. (B) The mRNA level of *Il-6* and *Il-8*. (C, E) Representative images are shown SA-β-gal staining (left), and percentages of SA-β-gal positive cells (right). All statistical data were calculated based on three independent experiments. The scale bar indicates 50 μm. * $p < 0.05$, ** $p < 0.01$, *** $p < 0.001$

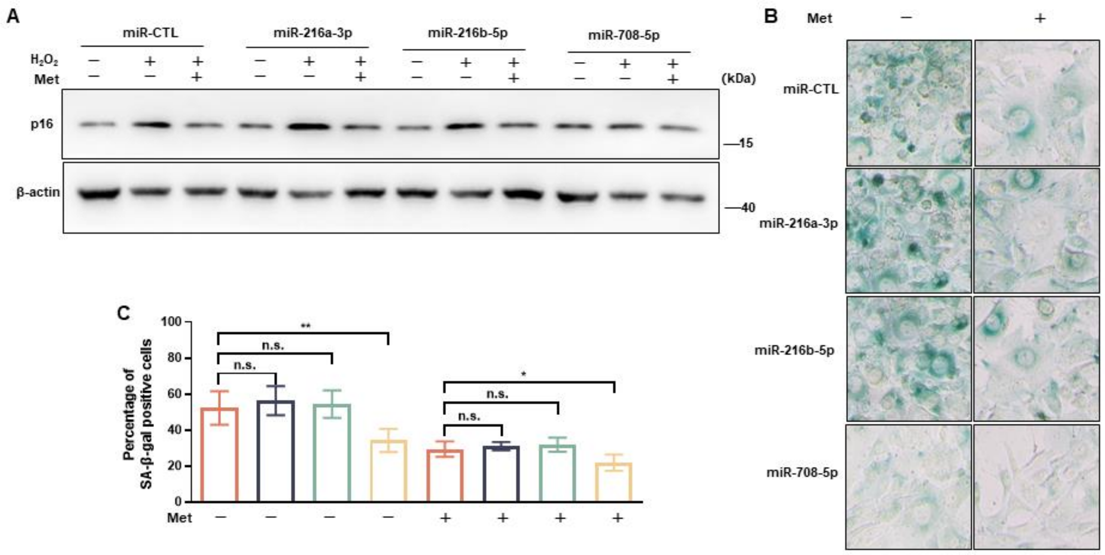

SUPPLEMENTARY FIGURE 2

Preliminary experiments of miRNAs overexpression affect cellular senescence. NIH3T3 cells were transfected with miR-216a-3p, miR-216b-5p, miR-708 mimics or scrambled miRNA (miR-CTL) for 24 h, followed by the H_2O_2-induced senescence with or without Met intervention. (A) Representative images are shown by immunoblotting assays against indicated p16. (B) Representative images are shown SA-β-gal staining. (C) Percentages of SA-β-gal positive cells. All statistical data were calculated based on three independent experiments. The scale bar indicates 50 μm. n.s. $p > 0.05$, * $p < 0.05$, ** $p < 0.01$

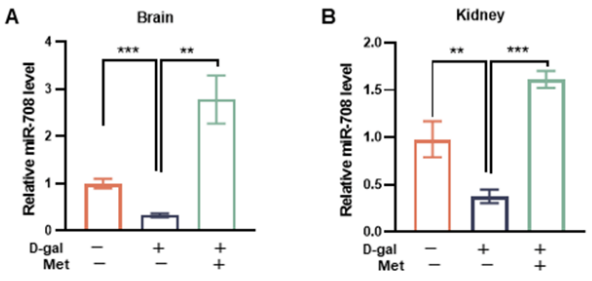

SUPPLEMENTARY FIGURE 3

Relative miR-708 level in mice. (A) Relative miR-708 level in brain of mice with or without D-gal- or Met-injection (n = 3). (B) Relative miR-708 level in kidney of mice with or without D-gal- or Met-injection (n = 3). ** $p < 0.01$, *** $p < 0.001$

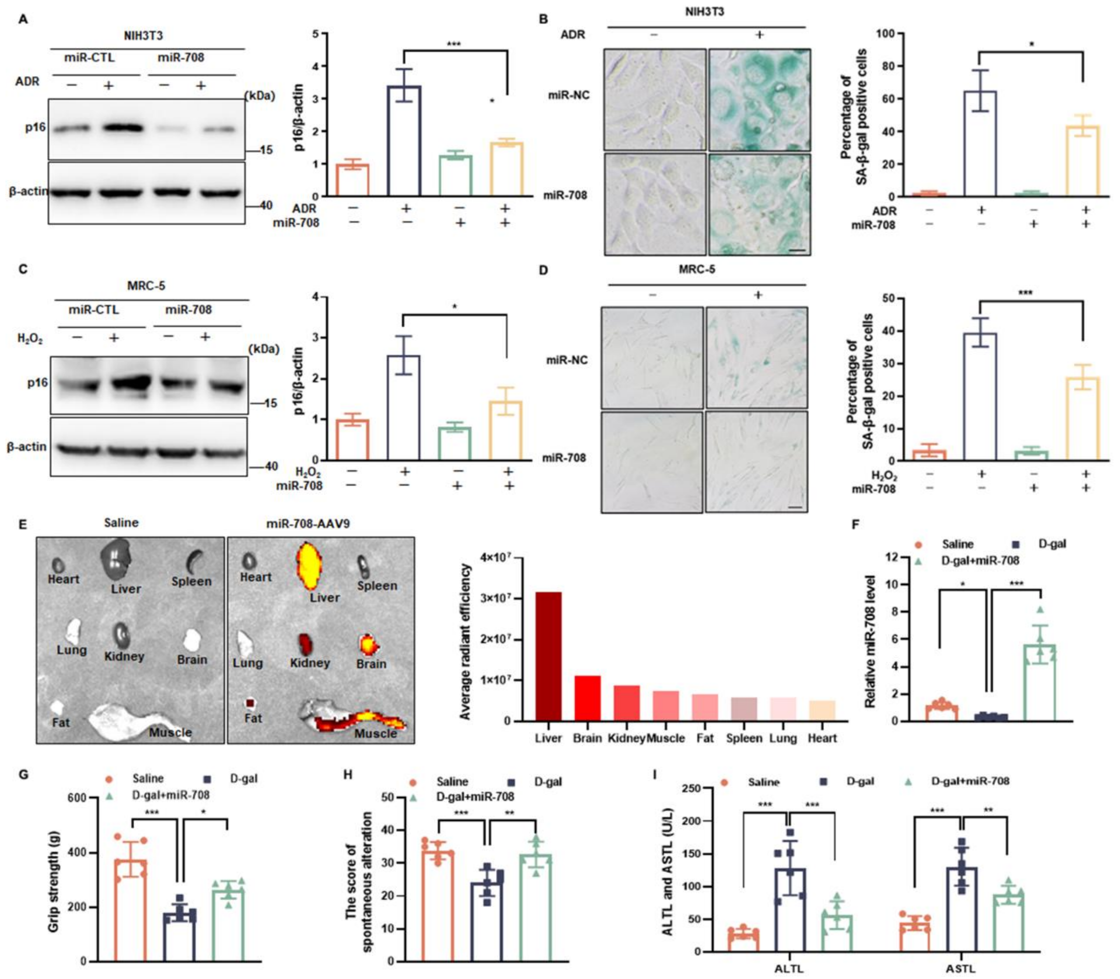

SUPPLEMENTARY FIGURE 4

miR-708 overexpression prevents aging. A, B: NIH3T3 cells were transfected with miR-708 mimics or scrambled miRNA (miR-CTL) for 24 h, followed by the senescence induction with ADR treatment. C, D: MRC-5 cells were transfected with miR-708 mimics or scramble miRNA (miR-CTL) for 24 h, followed by the senescence induction with H_2O_2 treatment. (A, C) Representative images from immunoblot assay (left), and the relative β-actin level normalized level of p16 protein (right). (B, D) Representative images from SA-β-gal staining (left), and percentages of SA-β-gal positive cells (right). (E) Representative fluorescent images of miR-708/GFP. (F) miR-708 expression level in liver. (G) Grip strength, measured by grip strength meter. (H) Score of spontaneous alteration, evaluated by Y-maze apparatus. (I) ALTL and ASTL(U/L) activities in serum. All cell culture experiments were repeated at least three times. Scale bar indicates 50 µm. * $p < 0.05$, ** $p < 0.01$, *** $p < 0.001$

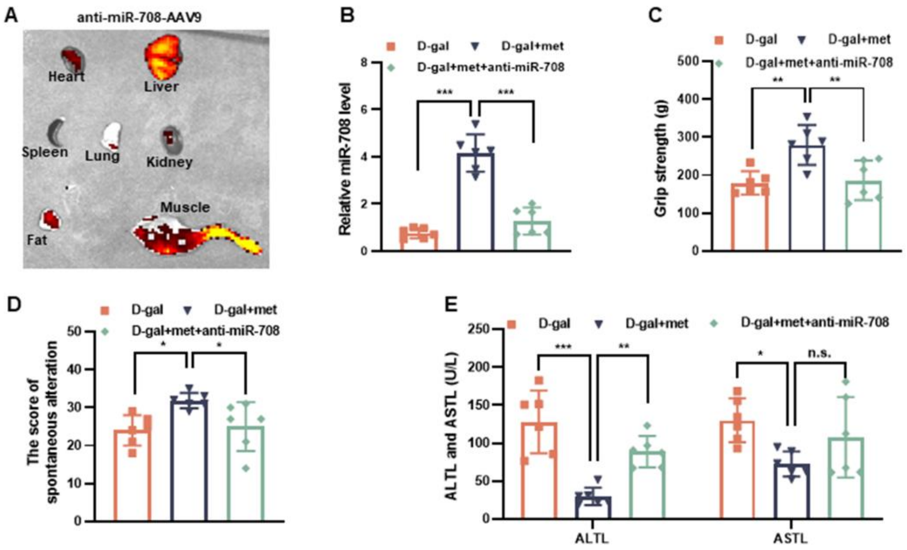

SUPPLEMENTARY FIGURE 5

miR-708 participates in the anti-aging effect of AMPK in mice. (A). Representative fluorescent images of anti-miR-708/mcherry. (B) miR-708 expression level in liver. (C) Grip strength, measured by grip strength meter. (D) Score of spontaneous alteration, evaluated by Y-maze apparatus. (E) ALTL and ASTL(U/L) activities in serum. n.s. $p > 0.05$, * $p < 0.05$, ** $p < 0.01$, *** $p < 0.001$
